# Restoration of a malformed primary incisor using digital technology in a pediatric patient with congenital Zika virus syndrome: A case report

**DOI:** 10.34172/joddd.2022.012

**Published:** 2022-05-29

**Authors:** Isabella Fernandes Carvalho, Louise Cristina Pereira Freitas, Phillipe Nogueira Barbosa Alencar, Maria Cláudia de Freitas Lima, Daniel de Sá Cavalcante, José Luciano Pimenta Couto, Paulo Tarcio Aded Silva, Dhaniel Anderson Olímpio Barbosa, Ellaine Dóris Fernandes Carvalho, Fabrício Bitu Sousa

**Affiliations:** ^1^Clinic School of Dentistry, Christus University Center, UNICHRISTUS, Brazil; ^2^Clinic School of Medicine, Christus University Center, UNICHRISTUS, Brazil; ^3^Clinic School of Dentistry, Federal University of Ceará, UFC, Brazil

**Keywords:** Dental anomalies, Indirect restorations, Oral manifestations, Zika virus

## Abstract

Zika virus congenital syndrome (ZVCS) is a congenital viral infection resulting from the transmission of the Zika virus (ZV) to the fetus during pregnancy. This report describes a clinical case involving a 20-month-old female child with ZVCS, who presented with systemic changes related to the syndrome, such as microcephaly, arthrogryposis, ocular and auditory changes, and oral changes such as delayed dental eruption, ogival (high-arched) palate, short lip frenum, and altered morphology of a superior primary incisor. For esthetic and functional rehabilitation of the oral health of this child, an indirect composite resin restoration was performed using intraoral digital scanning technology. This case presents an accurate, rapid, and comfortable restorative treatment option that might result in excellent outcomes in children with ZVCS or similar syndromes with neurological impairment.

## Introduction

 The Zika virus (ZV) originated from the African continent and was first seen in rhesus monkeys of the Zika Forest in Uganda in 1947.^1^ From 2007 onward, outbreaks of the virus occurred in the Pacific islands and then in French Polynesia in 2013.^2-4^

 In 2015, a major ZV outbreak occurred in Brazil, which coincided with an unusual increase in the number of newborns with severe brain abnormalities, with microcephaly being the most severe abnormality. Later, it was discovered that these changes were caused by deficiencies in cell proliferation and the death of neural progenitor cells infected by ZV, which could access the fetal brain during pregnancy by crossing the placental barrier. These findings validated the relationship of the virus with the significant increase in the number of newborns with microcephaly.^1^

 In addition to microcephaly, children born with ZV infections showed other alterations as well, including visual alterations, hearing impairment, arthrogryposis, skeletal deformities, epilepsy, dysphagia, and cognitive retardation, all of which were defined under the term Zika virus congenital syndrome (ZVCS).^5-7^

 Between the end of 2015 and the beginning of 2020, a total of 3512 confirmed cases of ZV infections were reported in newborns in Brazil. Although the number of cases has decreased considerably since the peak period, more than half a dozen new cases have occurred since 2018. Thus, although the emergency period has ended, new cases continue to appear in the country.^8^

 A study by Carvalho et al^9^ showed that children with ZVCS exhibited several changes in oral development during the first 24 months of life, such as a greater tendency for delayed eruption of the first deciduous tooth, inadequate lingual posture, narrow palate, and short labial and lingual frena. In addition, changes in the morphology, number, and sequence of tooth eruption have been reported. The changes in the dental development of children with ZVCS reported in the literature indicate the possible dental effects of the virus.^10^

 The dental morphology changes noted in some patients with the ZVCS require proper restoration to restore function and esthetics, and simple procedures with short execution times and prolonged durability should be used for this purpose.^11^ These restorative procedures include indirect restorations in acrylic resin and indirect and direct restorations in composite resin that might involve the use of the celluloid matrix. However, since indirect composite resin restorations offer good accuracy, lower treatment costs, and reduced clinical time, they are an excellent option for oral rehabilitation in patients with special needs, such as those with ZVCS.^11^

 For indirect restorations in composite resin, it is necessary to previously record the dental arches through conventional casting or digital scanning. The restoration is fabricated on the model obtained, which will then be adjusted and cemented directly on the tooth.^12^ On a related note, the use of intraoral scanners has emerged as a new technological trend in dentistry; in this approach, images are obtained through digital intraoral scanning (EDI) for the diagnosis and planning of clinical cases.^13,14^

 We report a clinical case in which a primary incisor with altered morphology in a patient with ZVCS was restored using an indirect restoration with a printed model after digital scanning.

## Case Report

**Figure 1:**
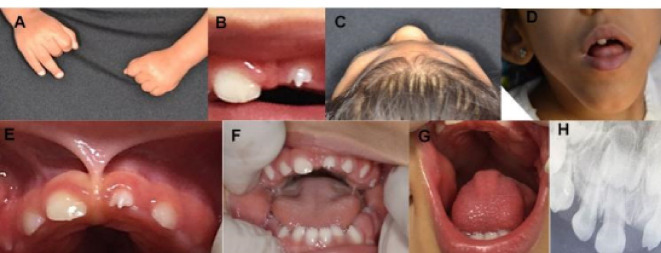
A) The marks and injuries caused by the deciduous maxillary left central incisor with the accessory cuspid left on the patient’s hands. B) Intraoral photo of the deciduous maxillary central incisors showing the change in dental shape in the deciduous maxillary left central incisor. C) Photograph of the upper view of the patient’s head showing microcephaly. D) Photograph of the frontal view demonstrating the absence of the lip seal and perioral hypotonia. E) Photograph showing the appearance of the anomaly and an ugly upper lip with low insertion. F) Intraoral photograph showing the ogival palate. G) Intraoral photograph demonstrating the other erupted teeth. H) Modified periapical radiograph demonstrating the shape change in the deciduous maxillary left central incisor relative to the deciduous maxillary right central incisor.

 A 20-month-old female patient with ZVCS presented with her mother at the School of Dentistry Clinic with a chief complaint of self-injury in both hands secondary to a “pointed tooth” (Figures 1A and 1B). The patient had microcephaly (Figure 1C), arthrogryposis, ocular and auditory alterations, and a history of anticonvulsant and antispasmodic muscle medication.

 Clinical examination of the patient revealed lip incompetence, hypotonia of the perioral structures (Figure 1D), an upper lip frenum with low insertion (Figure 1E), a deciduous maxillary left central incisor teeth with altered morphology (Figure 1B), ogival palate (Figure 1F), open bite, and a history of delayed tooth eruption. In addition, the presence of erupted healthy deciduous maxillary right canine, lateral incisor, central incisor, deciduous maxillary left lateral incisor, canine, first molar, deciduous mandibular left second molar, canine, lateral incisor, central incisor, deciduous mandibular right central incisor, lateral incisor, and canine (Figure 1G).

 A digitally modified periapical radiograph was obtained with a low radiation dose, which showed morphological changes in the crown of the deciduous maxillary left central incisor teeth compared to the deciduous maxillary right central incisor (Figure 1H); the deciduous maxillary left central incisor had a smaller clinical crown height, thin enamel, smaller mesial diameter, and a “claw” cusp. In addition, the normality of the other teeth and the presence of permanent tooth germs were visualized.

**Figure 2:**
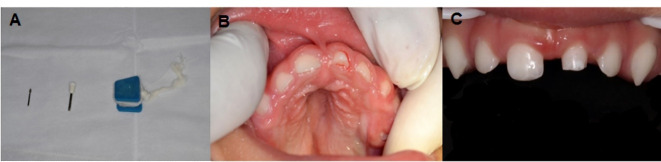
A) Worn-out cuspid. B) Armamentarium for selective grinding. C) Appearance after the total eruption of the deciduous maxillary left central incisor.

 The patient’s parents consented to dental treatment, which was performed in two stages to rehabilitate the deciduous maxillary left central incisor, since the tooth had not erupted completely. In the first stage, selective reshaping of the tip of the pointed cuspid was performed in a single session (Figure 2A). The procedure was performed using a flame-shaped diamond bur in a high-speed handpiece, followed by topical application of fluoride gel (FGM, Joinville, Brazil), using a P-size bite block to keep the mouth open during the procedure (Figure 2B).

**Figure 3:**
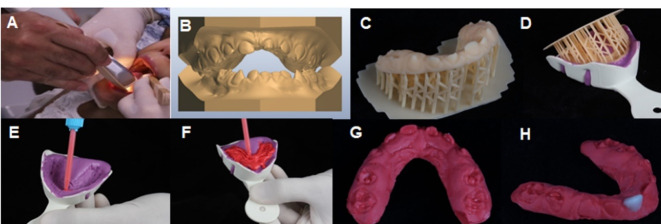
A) Intraoral scanning of the arch inferior. B) Computerized image resulting from intraoral scanning in the frontal view. C) 3D printing of the upper arch. D) Molding of the 3D model with alginate. E) Insertion of the light addition silicone impression material into the tray. F) Leakage of the mold with the light addition silicone impression material. G) Model made with light addition silicone impression material. H) Indirect restoration of composite resin for rehabilitation of the deciduous maxillary left central incisor.

 The patient was monitored periodically until complete eruption of the deciduous maxillary left central incisor (Figure 2C), which occurred at 36 months of age. Subsequently, the second stage of treatment, performed over two clinical sessions, was instituted by restoration using light-cured composite resin with the indirect technique and EDI using the TRIOS® scanner (3Shape A/S, Copenhagen, Denmark). The patient’s upper and lower arches were scanned (Figures 3 and 3B) without prior dental preparation for the deciduous maxillary left central incisor in a clinical session lasting 15 min. Despite the emergence of obstacles, such as the fact that the tip was large for the child’s oral cavity despite the need for it to remain immobile throughout the process, the procedure was successfully performed under rapid physical restraint, with the child lying on the mother’s lap and a size P bite block to maintain the oral opening. The patient was released and scheduled to undergo a second session that would involve the cementation of the indirect restoration.

 After scanning, a 3D impression of the upper dental arch (Figure 3C) was obtained using the Anycubic Photon X printer, and the impression was taken with an alginate Hydrogum 5 impression material (Zhermack, Badia Polesine (RO), Italy) (Figure 3D), followed by pouring the cast with light addition silicone impression material (Voco, Cuxhaven, Germany) (Figures 3E and 3F). Thus, the upper arch model was made in light addition silicone impression material (Figure 3G) on which the indirect composite resin restoration was manually fabricated to restore the deciduous maxillary left central incisor (Figure 3F). The restoration was fabricated using the incremental composite resin technique (3M, Two Harbors, United States) using a Suprafill spatula and respecting the color stratification.

**Figure 4:**
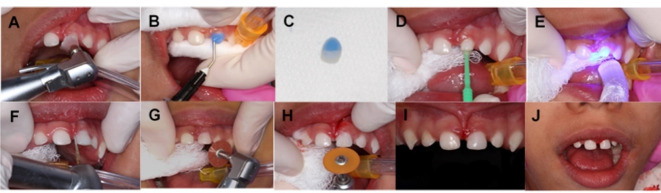
A) Prophylaxis with a pumice stone. B) Acid conditioning of the tooth. C) Acid conditioning of the part. D) Application of adhesive on the dental structure. E) Photopolymerization on all dental sides. F) Finishing and polishing the restoration using diamond tips. G) Finishing and polishing of the restoration using fine granulated Sof-lex discs. H) Finishing and polishing of the restoration using ultra-fine grain Sof-lex discs. I) Immediate result. J) Immediate result.

 The patient returned to the School of Dentistry Clinic for the cementation of the composite resin crown. The clinical protocol was started with the dental crown test to assess adaptation, after which prophylaxis was performed with a polishing brush and pumice stone (Figure 4A) and conditioning with 37% phosphoric acid (Maquira, Maringá, Brazil) in the dental structure of the deciduous maxillary left central incisor (Figure 4B) and in the composite resin crown (Figure 4C) for 30 s, in accordance with the manufacturer’s instructions. After rinsing the phosphoric acid with water, the adhesive Adapter Single Bond 2 (3M, Two Harbors, United States) (Figure 4D) was applied, followed by light-curing for 10 s on all the dental surfaces, in accordance with the manufacturer’s instructions (Figure 4E). Subsequently, the indirect restoration was cemented with RelyX^TM^ ARC (3M, Two Harbors, United States) resin cement, which was applied inside the restoration and fitted into the deciduous maxillary left central incisor, allowing the cement to flow over the entire margin of the restoration, and the excess cement was removed with an explorer probe. Subsequently, all the faces were photopolymerized for 40 s, in accordance with the manufacturers’ instructions. Finally, finishing and polishing were performed using diamond tips (Figure 4F) and fine or ultra-fine granulated Sof-lex discs (Figures 4G and 4H), followed by occlusal adjustment. The total clinical time for the cementation of the composite resin crown was 10 min, and the restorative treatment was completed in two clinical sessions, yielding a predictable result and ensuring the restoration of aesthetics and patient function (Figures 4I and 4J). After two weeks, the patient returned for the re-evaluation appointment, and the restoration was found to be intact and the parents were satisfied with the outcome.

## Discussion

 To the best of our knowledge, this is the first case wherein an indirect esthetic restoration was performed on a child with ZVCS. This approach was chosen mainly because this patient had special needs, and her clinical care was full of obstacles and required adaptation by the dentist. However, the American Academy of Pediatric Dentistry advocates that all individuals with special health needs should receive ideal care since ignoring oral problems can negatively affect the individual’s quality of life.

 Given the changes in oral development in children with ZVCS, such as a greater tendency for the delayed eruption of the first deciduous tooth and changes in the morphology, number, and sequence of tooth eruption, it is necessary to prepare professionals for the phenotypes related to the syndrome as well as appropriate treatments.^9,15,16^

 Considering the dental problems that children with the ZVCS might have, it is worth noting that, according to Inagaki et al^17^ and Blanco et al,^18^ changes in the morphology, number, and color interfere with smile harmony, especially when they occur in the anterior maxilla, which can cause esthetic problems, making it difficult for these children to socialize. Moreover, Kouri et al^19^ pointed out that although deciduous teeth have a limited time in the oral cavity, they are extremely important for chewing, phonation, and occlusion until their complete exfoliation. The importance of restoring primary teeth with morphological changes is thus emphasized to restore dental integrity, which is directly linked to the stomatological function and well-being of these individuals.

 Al-Batayneh et al^20^ showed that patients with a history of seizures have an increased dental trauma index; therefore, it is understood that these patients might have limited indications for extensive restorations, given the risk of fracture during seizures. However, although the patient in question had a history of seizures, they were controlled by medications. Furthermore, since the patient had an anterior open bite and, therefore, no contact with the antagonist in the region of the restored tooth, the risk of fracture of the restoration during an eventual seizure was ruled out.

 Indirect composite resin restorations are among the best resources for dental crown reconstruction. According to Garcia et al^21^ and Rank et al,^12^ these restorations offer the advantages of low cost, better bonding ability to dental structures, association with adhesive cementation, and the possibility of performing any repairs after cementation. In addition, Garcia et al^21^ noted that composite resins allow intraoral adjustments and polishing, promote less abrasion to the antagonist dentition, and are more affordable than some materials used for the same purposes, such as ceramics. For the rehabilitation of children and patients with special needs, restorations using the indirect composite resin technique are an ideal choice since, according to Rank et al,^12^ its preparation mode is in an extraoral environment, ensuring better precision of the technique and reduced chair time.

 According to Soares et al,^11^ the direct restorative technique with or without a celluloid matrix could also be used with good results, but Rank et al^12^ noted that this technique requires, in addition to more skill on the part of the professional, more time from the child in the dental chair and is thus indicated for patients who can cooperate with clinical treatment. Thus, Garcia et al^21^ and Loguercio et al^22^ reported the use of indirect composite resin restorations to reduce the failures that occur with the direct use of resins, with the advantage of complete polymerization of the material, which directly interferes with wear resistance, color, esthetics, and long-term polishing. Furthermore, indirect use allows the material to show better bonding property with the dental structures associated with adhesive cementation and the possibility of eventual repairs after cementation.

 According to Bósio et al,^13^ digital intraoral scanning is an excellent choice for obtaining models for indirect restorations in patients whose time of clinical care should be as short as possible. The authors also emphasize that scanners can improve the quality of the record and reduce the cost and time of obtaining images. In addition, EDI allows 3D images to be viewed on computer screens, reducing, in some cases, the need for physical space for storage of plaster models.

 Kravitz et al^23^ explained that conventional impressions with materials such as alginate cause discomfort and increase patient anxiety, especially in children and patients with special needs. Furthermore, Chalmers et al^14^ reported that plaster models are heavy, brittle, and bulky, causing difficulties in storage. Studies by Fleming et al^24^ and Roberta et al^25^ showed that digital models could replace plaster models without impairing any clinical outcome. In addition to not requiring space for storage, they would avoid any possibility of allergy or sensitivity to the molding material. Finally, the acquisition of records through scanning reduces the risks of cross-contamination and post-molding, which are extremely relevant given the importance of controlling the spread of microorganisms.

 Although EDI is minimally invasive and more comfortable than conventional molding, one of the few disadvantages of scanning is the need for the patient to remain as still as possible during the procedure. However, parental involvement and previous guidance can be used to achieve better results in this regard.

## Conclusion

 The use of the indirect restoration technique with composite resins associated with digital scanning proved an excellent option for rehabilitation treatment of teeth with morphological changes in patients with ZVCS, ensuring satisfactory results with functional and esthetic restorations, greater predictability of the results for the professionals, and the greatest possible comfort for the patient with special needs. Our case’s findings emphasize the need for such rehabilitation approaches and promote methods of excellence that can optimize and enable the care of patients with neurological needs equal or similar to those in ZVCS.

## Acknowledgments

 We would like to thank Editage (https://www.editage.com) for English language editing.

## Authors’ Contribution

 All the authors have made substantive intellectual contributions to a published study. IC, LF, PA, and FB initiated, conceptualized, supervised the clinical work, and revised it critically for important intellectual content. ML, DC, JLC, and PAS contributed to all the clinical, radiographic care, and oral digital scanning of the patient. DB and EC drafted and revisited the work.

## Funding

 This research did not receive any specific grant from funding agencies in the public, commercial, or not-for-profit sectors.

## Ethics Approval

 The study was approved by Christus University Center, UNICHRISTUS, Brazil approvall code: 60740616.4.00005049.

## Competing interests

 None.
